# Differentially Methylated Regions in Desmoid-Type Fibromatosis: A Comparison Between CTNNB1 S45F and T41A Tumors

**DOI:** 10.3389/fonc.2020.565031

**Published:** 2020-10-29

**Authors:** Milea J. M. Timbergen, Ruben Boers, Anne L. M. Vriends, Joachim Boers, Wilfred F. J. van IJcken, Marla Lavrijsen, Dirk J. Grünhagen, Cornelis Verhoef, Stefan Sleijfer, Ron Smits, Joost Gribnau, Erik A. C. Wiemer

**Affiliations:** ^1^ Department of Surgical Oncology, Erasmus MC Cancer Institute, University Medical Center Rotterdam, Rotterdam, Netherlands; ^2^ Department of Medical Oncology, Erasmus MC Cancer Institute, University Medical Center Rotterdam, Rotterdam, Netherlands; ^3^ Department of Developmental Biology, Oncode Institute, Erasmus MC, University Medical Center, Rotterdam, Netherlands; ^4^ Center for Biomics, Erasmus MC, University Medical Center, Rotterdam, Netherlands; ^5^ Department of Gastroenterology and Hepatology, Erasmus MC, University Medical Center, Rotterdam, Netherlands

**Keywords:** aggressive fibromatosis, β-catenin, DNA methylation, epigenomics, soft tissue sarcomas

## Abstract

**Introduction:**

The majority of desmoid-type fibromatosis (DTF) tumors harbor a β-catenin mutation, affecting specific codons in *CTNNB1* exon 3. S45F tumors are reported to have a higher chance of recurrence after surgery and more resistance to systemic treatments compared to wild-type (WT) and T41A tumors. The aim of this pilot study was to examine the genome-wide DNA methylation profiles of S45F and T41A mutated DTF, to explain the observed differences in clinical behavior between these DTF subtypes.

**Material and Methods:**

Genome-wide analysis of DNA methylation was performed using MeD-seq on formalin-fixed, paraffin-embedded primary DTF samples harboring a S45F (n = 14) or a T41A (n = 15) mutation. Differentially methylated regions (DMRs) between S45F and T41A DTF were identified and used for a supervised hierarchical cluster analysis. DMRs with a fold-change ≥1.5 were considered to be differentially methylated and differences between S45F and T41A tumors were quantitatively assessed. The effect of DMRs on the expression of associated genes was assessed using an independent mRNA expression dataset. Protein-protein interactions between WT β-catenin and mutant variants and DNA methyltransferase 1 (DNMT1) were examined by immunoprecipitation experiments.

**Results:**

MeD-seq analyses indicated 354 regions that displayed differential methylation. Cluster analysis yielded no distinct clusters based on mutation, sex, tumor site or tumor size. A supervised clustering based on DMRs between small (≤34 mm) and large (>87 mm) DTF distinguished the two groups. Only ten DMRs displayed a fold change of ≥1.5 and six of them were found associated with the following genes: *NLRP4*, *FOXK2*, *PERM1*, *CCDC6*, *NOC4L*, and *DUX4L6*. The effects of DMRs on gene expression yielded a significant difference (p < 0.05) in the expression between S45F and T41A for *CCDC6* and *FOXK2* but not for all Affymetrix probe-sets used to detect these genes. Immunoprecipitations did not reveal an association of WT β-catenin or mutant variants with DNMT1.

**Conclusion:**

This study demonstrated that S45F and T41A DTF tumors did not exhibit gross differences in DNA methylation patterns. This implies that distinct DNA methylation profiles are not the sole determinant for the divergent clinical behavior of these different DTF mutant subtypes.

## Introduction

Desmoid-type fibromatosis (DTF) is a rare, non-metastasizing, invasive, mesenchymal soft tissue sarcoma (1, 2). The presence of nuclear β-catenin distinguishes DTF from other soft tissue tumors and scar tissue (3) and is caused by the fact that the majority of tumors (>85%) harbor a mutation at specific sites in the β-catenin (*CTNNB1*) gene (4). These mutually exclusive mutations in exon 3 of the *CTNNB1* gene result in substitution of serine at position 45 with phenylalanine (S45F), or less commonly proline (S45P), or lead to a replacement of threonine at position 41 with alanine (T41A) (4, 5). The tumor is categorized as wild-type (WT) in case no *CTNNB1* exon 3 mutations are found. This WT group is considered heterogeneous as these tumors may contain other *CTNNB1*, outside of exon 3, or *APC* mutations (4). Despite the shared molecular basis in the majority of DTF patients, the clinical presentation and disease course varies. Several studies indicate a prognostic role for the *CTNNB1* mutation and some claim that S45F tumors have a higher risk of recurrence after surgery in comparison to T41A tumors (5–7) or that S45F tumors are more resistant to treatment with, e.g., sorafenib (8), doxorubicin (9), or meloxicam (10). Currently, there is no biological rationale for the reported clinical differences in behavior—particularly the risk of recurrence—of DTF with these mutation types. A recent pilot study revealed differences in the metabolomic profiles associated with T41A and S45F DTF cell lines also suggesting that—up to a certain extent—the biology of DTF with these *CTNNB1* mutations indeed differs (11).

The *CTNNB1* mutations that are predominantly observed in DTF prevent phosphorylation and subsequent degradation of β-catenin, a key player in the Wnt/β-catenin signaling pathway. This leads to stabilization and translocation of β-catenin into the nucleus, causing aberrant Wnt/β-catenin signaling. However, CTNNB1 has a complex role in the cell and is involved in protein-interaction networks related to cell adhesion and transcription. Nuclear β-catenin recruits transcription factors of the TCF family and interacts with epigenetic and chromatin modifiers (12, 13). Song et al. reported a protein interaction between CTNNB1 and the DNA methyltransferase DNMT1 in cancer cells which stabilizes each protein and regulates downstream CTNNB1 and DNMT1 functions suggesting a cross-regulation between Wnt signaling and DNA methylation (14).

Changes in the DNA methylation pattern have been described in various solid tumors, including various mesenchymal neoplasms such as chondrosarcoma, Ewing sarcoma, and rhabdomyosarcoma (15–20). These distinct methylation patterns could be of diagnostic value and capable of discerning tumor subtypes, may yield clinically relevant biomarkers that can direct treatment choices, and can potentially reveal novel treatment opportunities (21–25). The findings of Song et al. (14) prompted us to hypothesize that the different mutations found in CTNNB1 in DTF affect interacting DNMT1 differently consequently causing altered DNA methylation patterns.

This study investigates DNA methylation patterns of the two most common mutation types of DTF (S45F and T41A), aiming to provide insight in the biological underpinnings of the different clinical behavior of these DTF mutants.

## Materials and Methods

### Patient and Sample Selection

Patients with histologically proven, primary DTF and a S45F or T41A CTNNB1 mutation were identified in the Erasmus MC Pathology database. Corresponding formalin-fixed paraffin-embedded (FFPE) tumor tissue blocks were collected from the Erasmus MC tissue bank. Similarly, clinicopathological characteristics such as sex, age at diagnosis, tumor site (extra-abdominal, intra-abdominal, or abdominal wall), and largest tumor size [in millimeters (mm)] on imaging were obtained from the patient files. The *CTNNB1* exon 3 mutations were previously determined for diagnostic purposes essentially as described by Dubbink et al. (26) In short, tumor DNA was extracted from FFPE tumor tissue using proteinase K and 5% Chelex^®^-100 chelating resin (Bio-Rad). Sequence analysis of CTNNB1 exon 3 was performed by bidirectional sequencing of PCR-amplified DNA fragments using M13-tailed forward and reverse primers. The selected patients did not receive any treatment before the specimens were obtained. An expert soft tissue sarcoma pathologist confirmed the diagnosis by examining hematoxylin-eosin stained sections of the FFPE samples.

### DNA Isolation

DNA was isolated from five consecutive FFPE DTF sections of 10 µm using the Allprep DNA/RNA kit according to the manufacturer’s recommendations (Qiagen, Hilden, Germany). The DNA quality and the concentrations were determined using a Nanodrop-2000 (Isogen Life Science, Utrecht, The Netherlands). The 260 nm/280 nm ratio was ≥ 1.80 for all DNA preparations.

### MeD-Seq Sample Preparations

Methylated DNA sequencing (MeD-seq) was used to analyze genome-wide DNA methylation. MeD-seq provides single-nucleotide resolution by exploiting the properties of the DNA methylation dependent restriction enzyme *LpnPI* (27). This enzyme generates DNA fragments of 32 base pairs (bp) by cutting 16 bp downstream from the methylated CpG sites, which allows specific focus on the methylated regions. The MeD-seq analyses were essentially carried out as previously described (27, 28). In brief, DNA samples were digested by *LpnPI* (New England Biolabs, Ipswich, MA, USA), resulting in snippets of 32 bp around a fully-methylated recognition site that contains a CpG. These short DNA fragments were further processed using a ThruPlex DNA-seq 96D kit (cat no. R400407, Rubicon Genomics Ann Arbor, MI, USA) and a Pippin system. Stem-loop adapters were blunt-end ligated to repaired input DNA and amplified to include dual indexed barcodes using a high-fidelity polymerase to generate an indexed Illumina NGS library. The amplified end product was purified on a Pippin HT system with 3% agarose gel cassettes (Sage Science, Beverly, MA, USA). Multiplexed samples were sequenced on Illumina HiSeq2500 systems for single read of 50 bp according to the manufacturer’s instructions. Dual indexed samples were demultiplexed using bcl2fastq software (Illumina, San Diego, CA, USA).

### MeD-Seq Data Analysis

Data processing was carried out using specifically created scripts in Python. The proprietary Python script is used in the context of an exclusive license from the Erasmus Medical Center with Methylomics BV. Raw fastq files were subjected to Illumina adaptor trimming and reads were filtered based on *LpnPI* restriction site occurrence between 13 and 17 bp from either 5’ or 3’ end of the read. Reads that passed the filter were mapped to hg38 using bowtie2. Genome-wide individual *LpnPI* site scores were used to generate read count scores for the following annotated regions: transcription start sites [(TSS), 1 kb before and 1 kb after], CpG-islands and gene bodies [1 kb after TSS till Transcription End Site (TES)]. Gene and CpG-island annotations were downloaded from ENSEMBL (www.ensembl.org). Detection of DMRs was performed between two datasets containing the regions of interest (TSS, gene body or CpG-islands) using the Chi-square test on read counts. Significance at a p-value of <0.05 was called by either Bonferroni or FDR using the Benjamini-Hochberg procedure.

In addition, a genome-wide sliding window was used to detect sequentially differentially methylated *LpnPI* sites. Statistical significance was called between *LpnPI* sites in predetermined groups using the Chi-square test. Neighboring significantly called *LpnPI* sites were binned and reported. Annotation of the overlap of genome-wide detected DMRs was reported for TSS, CpG-islands and gene body regions. DMR thresholds were based on *LpnPI* site count. Fold-changes of read counts are mentioned in the figure legends before performing hierarchical clustering. The differentially methylated datasets generated and analyzed during the current study have been deposited to the Sequence Read Archive (SRA) data repository under accession number PRJNA604749. The DMRs with a fold-change ≥1.5 were considered to be differentially methylated and were analyzed separately. Non-normal distributed values were analyzed using a Mann-Whitney U test to identify statistically significant differences in the normalized read counts between the two mutation types. A p-value of < 0.05 was considered to be statistically significant. SPSS Statistics (version 24) was used for the Mann-Whitney U tests (IBM, Armonk, New York, USA). The DMRs of interest were loaded in the Integrative Genomics Viewer (IGV) using the Hg 38 platform to visualize regions of interest (29).

### Validation MeD-Seq Results Using an mRNA Expression Dataset

Expression data, generated on an Affymetrix platform (Human Genome U133 Plus 2.0 array) of *PERM1*, *DUX4L6*, *CCDC6*, *NOC4L*, *FOXK2*, and *NLRP4* in DTF samples (n = 128) were obtained from a publicly available dataset in the Gene Expression Omnibus (accession number GSE58697) (30) Information regarding the *CTNNB1* mutational status was kindly provided by Dr. Frederic Chibon, Cancer Research Center of Toulouse, France. Only patients with an S45F or T41A mutation were selected for validation purposes. A Mann-Whitney U test was performed on non-normal distributed data to identify differences in mRNA expression levels of the selected genes corresponding to the identified DMRs. A p-value of < 0.05 was considered to be statistically significant. SPSS Statistics (version 24) was used for all statistical analyses.

### Cell Lines, Cell Transfection

The human cell lines HEK293T (embryonic kidney cells) and HCT116 (colon cancer cells) were maintained in DMEM (Gibco Life Technologies) supplemented with 10% fetal bovine serum (Greiner bio-one) and 100 IU/ml penicillin and 100 μg/ml streptomycin at 37°C in a humidified atmosphere containing 5% CO2. For transfections cells were cultured in 6-well plates to 70% confluence. Next, the cells in each well were transfected with 1 µg of different N-terminal FLAG tagged β-catenin plasmids or empty pcDNA 5’UT-FLAG vector using ViaFect™ (Promega) as transfection agent. The construction of the different expression plasmids is described by Liu et al. (31). The CTNNB1 plasmid variants used, express FLAG tagged versions of either the WT, T41A, S45P, exon 3 deletion, or K335I β-catenin.

### Cell Lysates, Immunoprecipitation, and Western Blotting

At 48-h post-transfection, cells were washed with ice-cold PBS and lysed in 500 µl of lysis buffer 25 mM Tris-HCl pH 7.5, 150 mM NaCl, 1 mM EDTA, 1% NP-40, and 5% glycerol (Pierce IP lysis buffer) containing Halt Protease and Phosphatase inhibitor single-use cocktail (ThermoFisher Scientific). Wells were cleaned by scraping and the cell lysates collected and centrifuged at 11.000 × g for 10 min at 4°C to pellet insoluble cell debris. From the cleared lysates 10% was used as input control which is prepared for SDS-PAGE by adding an equal volume of 2× Laemmli sample buffer with 0.1 M DTT (Laemmli/DTT). FLAG-tagged β-catenin is immunoprecipitated from the remainder of the lysates using prewashed ANTI-FLAG M2 Affinity Gel (Sigma-Aldrich, cat. No. A2220) for 2 h at 4°C. FLAG-beads were washed with lysis buffer for three times and resuspended in Laemmli/DTT.

Input and IP samples were heated to 95°C for 5 min and subjected to SDS-PAGE and electroblotted onto polyvinylidene difluoride (PVDF) membranes. Membranes were blocked in TBS/0.1% Tween’20 supplemented with 5% (w/v) BSA and incubated overnight at 4°C with rabbit monoclonal anti-DNMT1 (1:1,000 DNMT1 XP^®^, D63A6, Cell Signaling Technology), mouse monoclonal anti-FLAG^®^ M2 antibody (1:1,000 Sigma-Aldrich, cat no. F1804) or mouse monoclonal anti-β-actin (1:10,000, Sigma-Aldrich, cat no. A5441). The primary antibodies were diluted in blocking buffer. HRP conjugated goat-anti-rabbit, goat-anti-mouse were used as secondary antibodies in TBS/0.1% Tween’20 supplemented with 5% (w/v) non-fat dried milk. Enhanced chemiluminescence (SuperSignal™ West Pico Plus Chemilumininescent Substrate, Thermo Scientific) was used to visualize the bound antibodies in a ChemiDoc MP Imager (Bio-Rad).

### Ethical Approval

This study was part of a protocol entitled “Translational research on soft tissue sarcomas” which was assessed by the Medical Ethics Committee of the Erasmus MC, Rotterdam, The Netherlands (MEC-2016-213).

## Results

### Clinical Characteristics of the Patients Included in the MeD-Seq Analysis

The vast majority of DTF tumors contain mutations in exon 3 of the *CTNNB1* (β-catenin) gene. Interestingly, the mutations are almost exclusively confined to residues T41 and S45 preventing the phosphorylation of these residues and consequently stabilizing *CTNNB1* and activating Wnt/β-catenin signaling. Although having similar effects, the T41A and S45F mutated DTF tumors were reported to display a different clinical behavior for which the underlying biological mechanism is still unclear. Epigenetic alterations may be important in this respect, particularly in view of the reported interaction between CTNNB1 and DNMT1 (14). Therefore, the genome-wide DNA methylation profiles of DTF tumors were explored, comparing CTNNB1 S45F and T41A mutated tumors. To this end, 29 FFPE samples of primary untreated DTF tumors were analyzed using MeD-seq. Fifteen samples harbored a CTNNB1 T41A mutation and 14 samples a CTNNB1 S45F mutation, both mutations that are commonly observed. The patients had a median age of 36 years (interquartile range 26–47 years) and females were in the majority (n = 19, 66%). Most tumors were located extra-abdominally (69%) and had a median size of 55 mm (interquartile range 34–87 mm) (**Table 1**).

**Table 1:** Clinical characteristics of patients and tumor samples (n = 29) included in this study.

		Total group n = 29	T41A n (%)	S45F n (%)
Sex	Female	19 (66%)	11 (73%)	8 (57%)
	Male	10 (34%)	4 (27%)	6 (43%)
Tumor location	Extra-abdominal	20 (69%)	7 (47%)	13 (93%)
	Abdominal wall	6 (21%)	5 (33%)	1 (7%)
	Intra-abdominal	3 (10%)	3 (20%)	0
Median age at diagnosis in years (IQR)		36 (26–47)	38 (33–48)	31 (20–45)
Median size in mm (IQR) ^a,b^		55 (34–87)	53 (29–59)	68 (50–103)

^a^Based on initial imaging; ^b^n = 3 missing values; IQR, interquartile range.

### Differentially Methylated Regions Between S45F and T41A DTF Samples Identified by MeD-Seq

**Figure 1:**
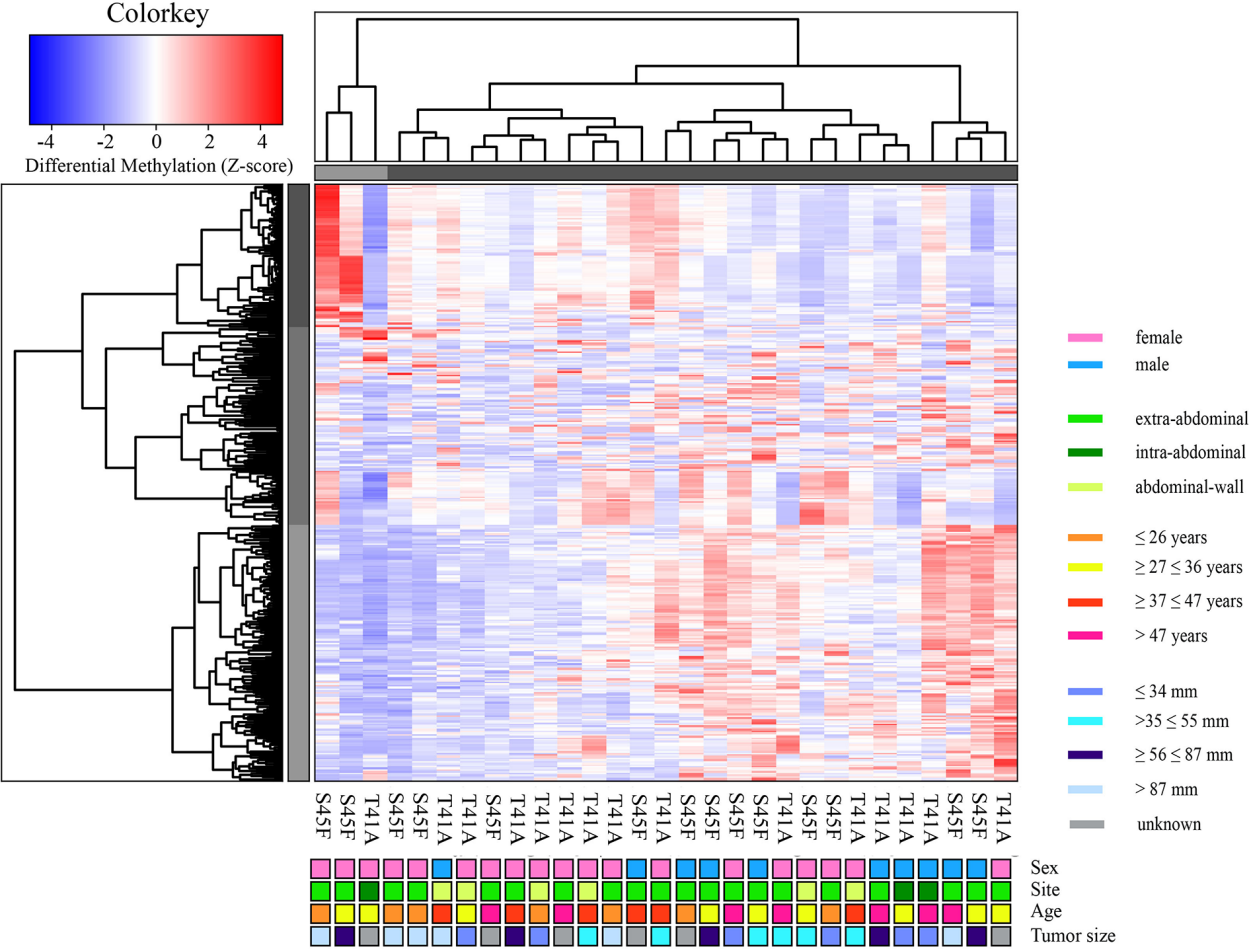
Supervised hierarchical clustering based on differentially methylated regions (DMRs) between S45F and T41A mutated DTF tumors together with clinicopathological features: sex, tumor site, age, and tumor size. The heat map depicts the methylation in 354 DMRs, including all fold-changes and excluding DMRs present on sex chromosomal regions, in S45F and T41A DTF samples. The cluster tree on top indicates distinct subgroups of DTF samples. Grouping, however, is not based on *CTNNB1* mutation type (T41A or S45F) nor on clinicopathological parameters such as sex, tumor site, age, or tumor size.

A genome-wide MeD-seq analysis was carried out using DNA isolated from 29 FFPE DTF samples. A total of 365 differentially methylated regions (DMRs) were found to be significantly different between S45F and T41A DTF tumors. After excluding DMRs located on the X and Y chromosomes, a group of 354 DMRs remained. Of these 354 DMRs, 97 (27%) DMRs were hypomethylated in S45F (vs. T41A), and 257 (83%) were hypermethylated in S45F (vs. T41A). **Supplemental File 1** provides an overview of all 354 DMRs. A supervised hierarchical clustering of the DTF samples based on all detected DMRs did not reveal clearly separated groups based on *CTNNB1* mutational status (**Figure 1**). Likewise, no distinct cluster patterns were observed on basis of sex (**Figure 1**), tumor site (**Supplemental Figure 1**), and tumor size (**Supplemental Figure 2**). As a meta-analysis by our group revealed tumor size to be an independent predictor for recurrence (6) a supervised cluster analysis was performed based on DMRs between small DTF (n = 6, ≤ 34 mm) and large tumors (n = 6, > 87 mm). Interestingly, the small and large DTF tumors were almost completely distinguished only one small tumor grouped together with the large tumors (**Supplemental Figure 3**).

**Figure 2:**
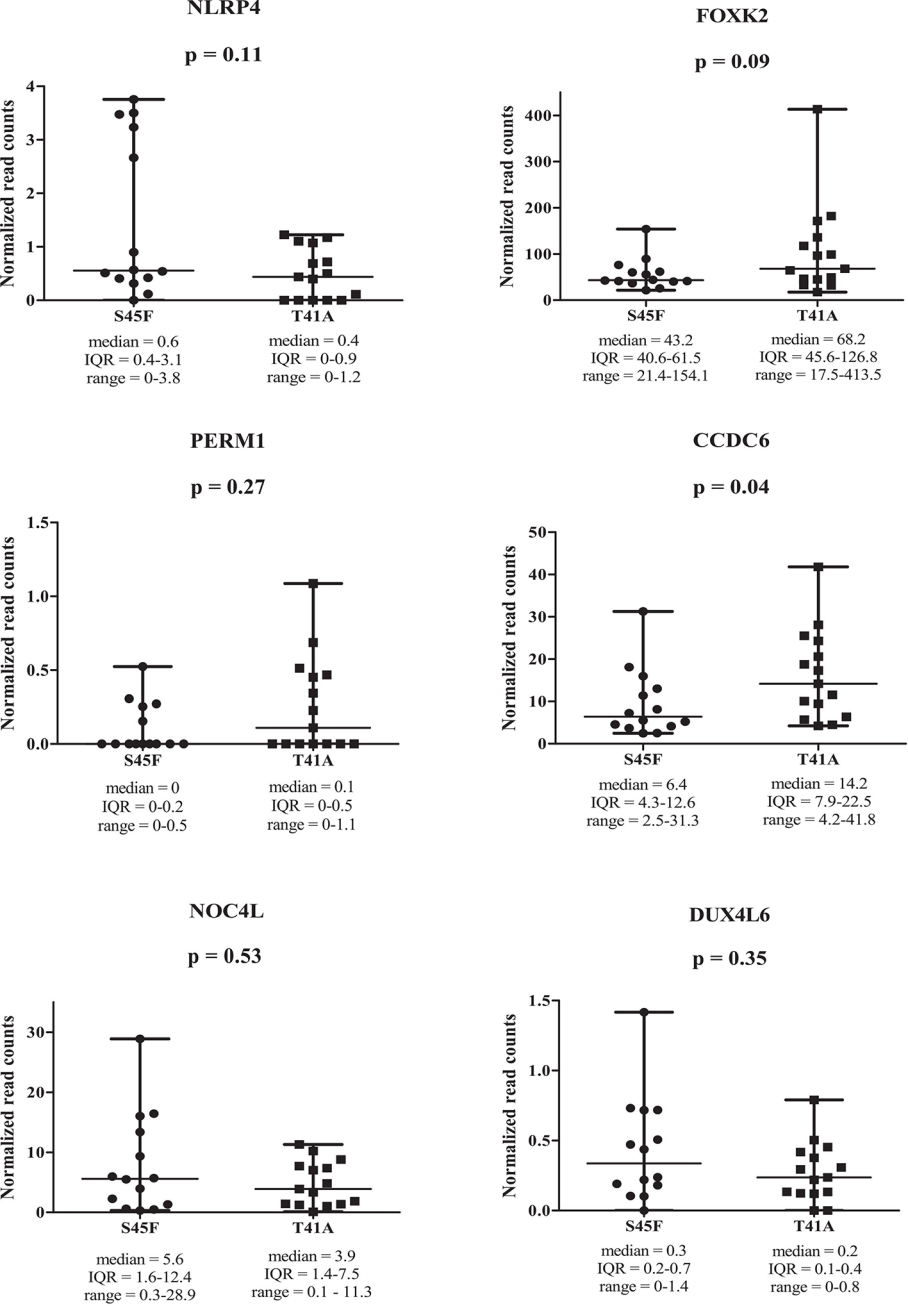
Read counts for DMRs in individual S45F and T41A DTF. Plots showing the normalized read counts for selected DMRs (fold-change ≥1.5) associated with *NLRP4*, *FOXK2*, *PERM1*, *CCDC6*, *NOC4L* and *DUX4L6*. The whiskers represent the minimum and maximum read counts. Dots (S45F) or squares (T41A) indicate individual data points, the horizontal line designates the median level. A Mann Whitney U test was used to assess statistical significance.

Within both CTNNB1 mutation groups, there appeared to be a considerable heterogeneity in DNA methylation between tumor samples. When considering all 354 DMRs it was noticed that the vast majority displayed relatively small fold-changes (<1.5) between the different DTF mutant groups. Only ten DMRs had fold-changes ≥1.5. **Table 2** lists the chromosomal position of the DMRs with a fold-change ≥1.5 including the start and end positions, the observed fold-change, the overlapping genes associated with the DMR, the location of the DMR with respect to the gene body and the methylation status in the S45F and T41A samples. Most genes, with the exception of *DUX4L6*, present with DMRs in the gene body. The DMRs and their location were visualized by loading the MeD-seq data into the Integrative Genomics Viewer (IGV) (**Supplemental Figure 4**). The IGV graphs present the methylation patterns in a quantitative way in each DTF sample that was analyzed. It was observed that the methylation profiles within a *CTNNB1* mutant class may differ considerable, with samples displaying almost a complete absence of methylation, whereas other samples appear (partly) methylated. **Figure 2** depicts the actual normalized read counts of the DMRs and associated genes detected between S45F and T41A DTF samples. A more stringent statistical analysis of the raw data revealed that only the DMR associated with *CCDC6* remained statistically significant (p = 0.04).

**Table 2:** Overview of DMRs with a fold-change ≥ 1.5 between S45F and T41A DTF.

Chromosome	Fold change	Position(start–end)	Methylation status		Overlapping genes	Position
			S45F	T41A		
19	2.68	55850028–55850071	+	−	NLRP4	postTSS1KB-TES
17_GL383563v3_alt	2.27	130411–131133	−	+	–	
17	1.85	82585430–82587461	−	+	FOXK2	postTSS1KB-TES
16	1.79	85410202–85410284	+	−	–	
1	1.77	977947–977974	−	+	PERM1	postTSS1KB-TES
21		42956670–42958541	+	−	–	
10	1.70	59882022–59883084	−	+	CCDC6	postTSS1KB-TES
10	1.69	42089321–42090999	+	−	–	
12	1.65	132149685–132150607	+	−	NOC4L	postTSS1KB-TES
4	1.50	190075267–133745860	+	−	DUX4L6	TSS

DMRs located on sex (X and Y) chromosomes were excluded; TSS, Transcription Start Site; TES, Transcription End Site; postTSS1KB-TES, indicates the region starting at 1 Kb after the TSS till the TES thus corresponding to the gene body without promoter region. −, hypomethylation; +, hypermethylation.

### Effects of DMRs on Gene Expression Levels

Next, the effect of the initially detected DMRs with a fold-change ≥ 1.5 on the expression of the associated genes was examined. DMR associated genes *NLRP4*, *FOXK2*, *PERM1*, *CCDC6*, *NOC4L*, and *DUX4L6* were identified in a publicly available mRNA expression dataset of 34 S45F and 45 T41A DTF samples. It was noted that the same genes are detected by multiple probes on the Affymetrix platform used (**Table 3**). A Mann-Whitney U test indicated that no significant expression differences were observed between S45F and T41A mutant DTF for most genes with the exception of *CCDC6* (p = 0.034, 1 out of 2 probes) and *FOXK2* (p = 0.004, 1 out of 4 probes) (**Table 3**). It was noted that the expression of *CCDC6* and *FOXK2* in T41A samples was downregulated suggesting that the hypermethylation observed in T41A samples reduces mRNA expression.

**Table 3:** Expression levels of genes identified by DMRs between S45F and T41A DTF samples.

Gene name	Probe number	Median (IQR)	p-value
CCDC6	204716_at	203 (126–239)	**0.034**
	225010_at	617 (554.6–703.4)	0.579
FOXK2	242937_at	17 (15–21)	**0.004**
	242938_s_at	34 (32–39)	0.384
	226224_at	149 (141–158)	0.373
	203064_s_at	112 (101–127)	0.533
DUX4L6	216472_x_at	11 (8–14)	0.362
	208201_at	15 (11–20)	0.510
NLRP4	242334_at	2.7 (1.2–4.2)	0.149
NOC4L	218860	50 (44–55)	0.628
PERM1	224501_at	1 (1–1)	0.415

Transcript levels are derived from publicly available Affymetrix-based mRNA expression data (GSE58697) of 45 T41A and 34 S45F tumors. Some genes are represented by multiple probe sets. Mann-Whitney U test was used to assess statistical significance in transcript levels of the respective genes between S45F and T41A tumors. Bold numbers indicate significant p values.

### CTNNB1(β-Catenin)—DNMT1 Protein Interaction

**Figure 3:**
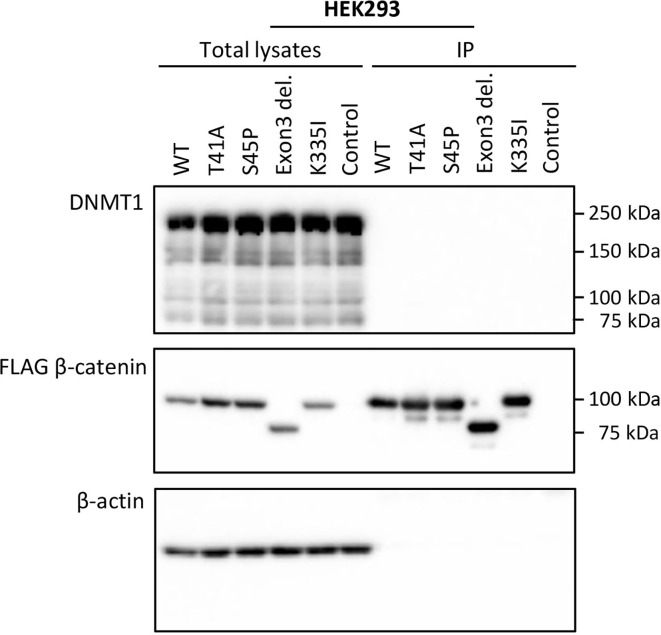
DNMT1 is not co-precipitated with wild-type or mutant CTNNB1 (β-catenin). HEK293T cells were transfected with plasmids driving the expression of FLAG-tagged wild-type β-catenin (WT) or FLAG-tagged mutant versions of β-catenin (T41A; S45P; Exon 3 deletion mutant; K335I). As a control cells were transfected with the empty vector. At 48-h post-transfection, cell lysates were prepared from which the FLAG-tagged β-catenin variants were immunoprecipitated. Western Blot analysis was used to examine DNMT1, β-catenin and β-actin protein expression in the total lysates and immunoprecipitates.

To verify whether the observed DMRs between S45F and T41A DTF samples could be the result of a differential regulatory interaction between β-catenin mutants and DNMT1, an immunoprecipitation experiment was performed. FLAG-tagged WT β-catenin and different β-catenin mutants (T41A, S45P, exon 3 deletion mutant, K335I) were transiently expressed in HEK293T and HCT116 cells. Western blot analysis of total lysates of the transfectants demonstrated expression of DNMT1 as well as the FLAG-tagged version of β-catenin and β-actin as loading control (**Figure 3** and **Supplemental Figure 5**). When FLAG-tagged β-catenin was immunoprecipitated no DNMT1 was co-precipitated. We tentatively conclude that we cannot verify a protein-protein interaction between β-catenin WT or mutants and DNMT1.

## Discussion

DNA methylation patterns are a good representation and reflection of molecular changes in the early stages of human cancer. The correlation between cancer and aberrant methylation patterns is described by various studies (21, 22, 25). DNA hypomethylation of gene promoter regions is usually associated with tumor formation, activation of oncogenes, and chromosomal instability (32, 33). In addition, DNA hypermethylation of gene promoter regions may alter gene expression and can cause tumor suppressor silencing and promote cancer progression (34). Also, gene body methylation is a widespread phenomenon; however, its functional consequences are less clear (35, 36). There is experimental evidence that gene body methylation is also associated with transcriptional activity and can affect gene expression (37, 38). Aberrant methylation has been the subject of various sarcoma-related publications. A study by Röhrich et al. based the classification of peripheral nerve sheath tumors (benign versus malignant) on methylation patterns (39). Tombolan et al. were able to distinguish metastatic and non-metastatic rhabdomyosarcomas based on their methylation profiles (17).

In this study, we hypothesized a role for aberrant methylation patterns based on the differences in clinical behavior between the different *CTNNB1* mutations found in DTF. To our knowledge, this is the first study that explored DNA methylation patterns in DTF. Whole-genome DNA methylation was examined using MeD-seq. This is a novel and powerful technique to perform genome-wide DNA methylation analyses (27). Since this technique focusses the sequencing resources on methylated regions only, and because the restriction enzyme *LpnPI* is restricted by a short template size, the generated fragments are consistent in size which results in accurate identification of DMRs genome-wide (27). The MeD-Seq method compared very favorable to other methods such as whole-genome bisulfite sequencing, MeDIP and the 450 K Infinium bead-chip technology (27). Furthermore, MeD-seq uses single base pair resolution which allows us to identify methylation on one specific CG site. In case of marker development, this would create the opportunity to use information from this single nucleotide for primer selection.

Here we focused on differences between *CTNNB1* T41A and S45F mutated tumors as they occur frequently, are mutually exclusive and are reported to exhibit a divergent clinical behavior. Overall, the detected differences in DNA methylation were few and subtle and unable to discriminate between S45F and T41A tumors in a cluster analysis. Only some DMRs were found to be differentially methylated with a fold-change ≥ 1.5, and only a single DMR, related to the *CCDC6* gene had a fold-change of ≥ 2. Most of the DMRs (fold-change ≥ 1.5) appeared to be situated within gene bodies. The relatively small fold-changes in DMRs observed, the intertumoral heterogeneity and the low amount of statistically significant DMRs identified in this study, suggests that there is no distinct difference in DNA methylation patterns between S45F and T41A DTF tumors. In the current study, the effects of differential methylation on gene expression were assessed using an independent Affymetrix mRNA expression dataset which only revealed significant expression differences between S45F and T41A tumors for *CCDC6* (p = 0.034) and *FOXK2* (p = 0.004) but not for all probes capable of detecting these genes. To explain these observations, one may speculate that gene body methylation affects differential splicing yielding splice products that hybridize only with some capture probes. To obtain biological insight why different β-catenin mutants would affect DNA methylation differently, the interaction between β-catenin and DNMT1, as reported by Song et al., was investigated (14). Despite the use of similar immunoprecipitation conditions and an identical cell line (HCT116) DNMT1 was not pulled-down with WT β-catenin or any of the β-catenin mutants tested.

No distinct cluster patterns were seen based on tumor size when a hierarchical clustering was performed using all 354 DMRs between S45F and T41A samples. Interestingly, when only the smallest and largest tumors were considered, DNA methylation patterns almost perfectly discriminated the two groups. Although tumor size depends on the measuring methods (radiological imaging or the dimensions of freshly resected surgical specimen) and can therefore be fairly subjective, our data suggest larger tumors display a different methylation pattern compared to smaller tumors. The phenomena that methylation patterns differ between tumor sizes has previously been described by Nishida et al., and may suggest that stepwise progression of methylation alterations may take place during the development of tumors (40).

This study has several limitations. The first limitation is the relatively small DTF sample size. DTF samples are challenging to obtain due to the rarity of these tumors and the current tendency to use an active surveillance approach instead of surgical resection (41). Furthermore, obtaining paired control tissue such as fascia from which desmoids are believed to arise, is challenging as it would require an additional resection of adjacent fascia next to the tumor site. Due to the retrospective nature of the current study, we were not able to obtain paired control tissue samples. Furthermore, we opted not to include WT DTF samples as control as they comprise a heterogeneous group which often contains rare *CTNNB1* mutations or alterations in other genes (4).

Future research should focus on the integrated genomic and molecular characterization of DTF samples and include appropriate control tissues to further delineate and understand the biological mechanisms and epigenetic changes involved in the pathogenesis of DTF. The functional significance of the observed differential gene body methylation of *CCDC6* in T41A and S45F DTF should be further validated and investigated. Exploration of the dynamic changes in DNA methylation patterns and their consequences for gene expression from tumor onset to tumor progression and/or regression is of interest too and may provide an explanation for the different clinical behaviors of DTF tumors.

## Data Availability Statement

The datasets presented in this study can be found in online repositories. The names of the repository/repositories and accession number(s) can be found below: Sequence Read Archive (SRA) data repository (www.ncbi.nlm.nih.gov/bioproject/PRJNA604749/), PRJNA604749.

## Ethics Statement

This study, part of a protocol entitled “Translational research on soft tissue sarcomas”, was reviewed and approved by the Medical Ethics Committee of the Erasmus MC, Rotterdam, The Netherlands (MEC-2016-213). Written informed consent for participation was not required for this study in accordance with the national legislation and the institutional requirements.

## Author Contributions

Conceptualization: MT, DG, CV, SS, and EW. Methodology: MT, RB, AV, JB, WIJ, and ML. Data curation: RB, JB, JG, RS, and EW. Formal analysis: RB, JB, and JG. Project administration: EW. Software: RB, JB, and JG. Supervision: DG, CV, SS, and EW. Validation: RB, JB, JG, RS, and EW. Visualization: MT. Writing—original draft: MT. Writing—review and editing: MT, RB, AV, JB, WIJ, ML, DG, CV, SS, RS, JG, and EW. All authors contributed to the article and approved the submitted version.

## Conflict of Interest

RB, JB, WIJ, and JG report being shareholders in Methylomics B.V., a commercial company that applies MeD-seq to develop methylation markers for cancer staging.

The remaining authors declare that the research was conducted in the absence of any commercial or financial relationships that could be construed as a potential conflict of interest.
