# Supplementary material for: Differentially Methylated Regions in Desmoid-Type Fibromatosis: A Comparison Between CTNNB1 S45F and T41A Tumors
Source: Front Oncol. 2020 Oct 29;10:565031. doi: 10.3389/fonc.2020.565031 (PMC7658920; doi:10.3389/fonc.2020.565031)
Supplement: Supplementary file 1 [file DataSheet_1.pdf]

Supplemental Information

Differentially methylated regions in desmoid-type fibromatosis: a comparison between CTNNB1 S45F and T41A tumors

Milea J.M. Timbergen, Ruben Boers, Anne L.M. Vriends, Joachim Boers, Wilfred F.J. van IJcken, Marla Lavrijsen , Dirk J. Grünhagen, Cornelis Verhoef, Stefan Sleijfer, Ron Smits, Joost Gribnau, Erik A.C. Wiemer

Supplemental Table 1 – List of 354 differentially methylated regions between S45F and T41A DTF samples. Excell sheet summarizing information on the detected DMRs including chromosomal localization, fold-change, position, methylation status in S45F and T41A DTF samples (+ hypermethylation; - hypomethylation), overlapping genes and position on the gene. TSS, Transcription Start Site; TES, Transcription End Site; postTSS1KB-TES, indicates the region starting at 1 Kb after the TSS till the TES thus corresponding to the gene body without promoter region.

Supplemental Figure 1 - Supervised hierarchical clustering based on differentially methylated regions (DMRs) between DTF tumor locations. DTF samples were obtained from tumors at extra-abdominal sites, intra-abdominal sites or the abdominal wall.

Supplemental Figure 2 - Supervised hierarchical clustering based on differentially methylated regions (DMRs) between DTF tumor sizes. Tumor sizes were based on initial imaging data obtained at diagnosis. The following size classes were analyzed ≤ 34 mm; >35mm ≤ 55 mm; ≥56 mm ≤ 87 mm; > 87 mm.

Supplemental Figure 3 - Supervised hierarchical clustering based on differentially methylated regions (DMRs) between DTF tumor size extremes. Tumor sizes were based on initial imaging data obtained at diagnosis. The smallest tumors $\leq$ 34 mm were compared to the largest tumors > 87 mm.

Supplemental Figure 4 A-F - Quantitative visualization of the differentially methylated regions with a fold-change ≥ 1.5 between S45F and T41A DTF. The DMRs are associated with genes: NLRP4, FOXK2, PERM1, CCDC6, NOC4L and DUX4L6. In the top panel, the chromosome involved with the location of the gene indicated by a small red block is depicted. The panels below represent the read counts found in either the S45F or T41A samples with the red box designating the location of the DMR.

Supplemental Figure 5 - DNMT1 is not co-precipitated with wild-type or mutant CTNNB1 (β-catenin). HCT116 cells were transfected with plasmids driving the expression of FLAG-tagged wild-type β-catenin (WT) or FLAG-tagged mutant versions of β-catenin (T41A; S45P; Exon 3 deletion mutant; K335I). As a control cells were transfected with the empty vector. At 48 h post-transfection cell lysates were prepared from which the FLAG-tagged β-catenin variants were immunoprecipitated. Western Blot analysis was used to examine DNMT1, β-catenin and β-actin protein expression in the total lysates and immunoprecipitates.

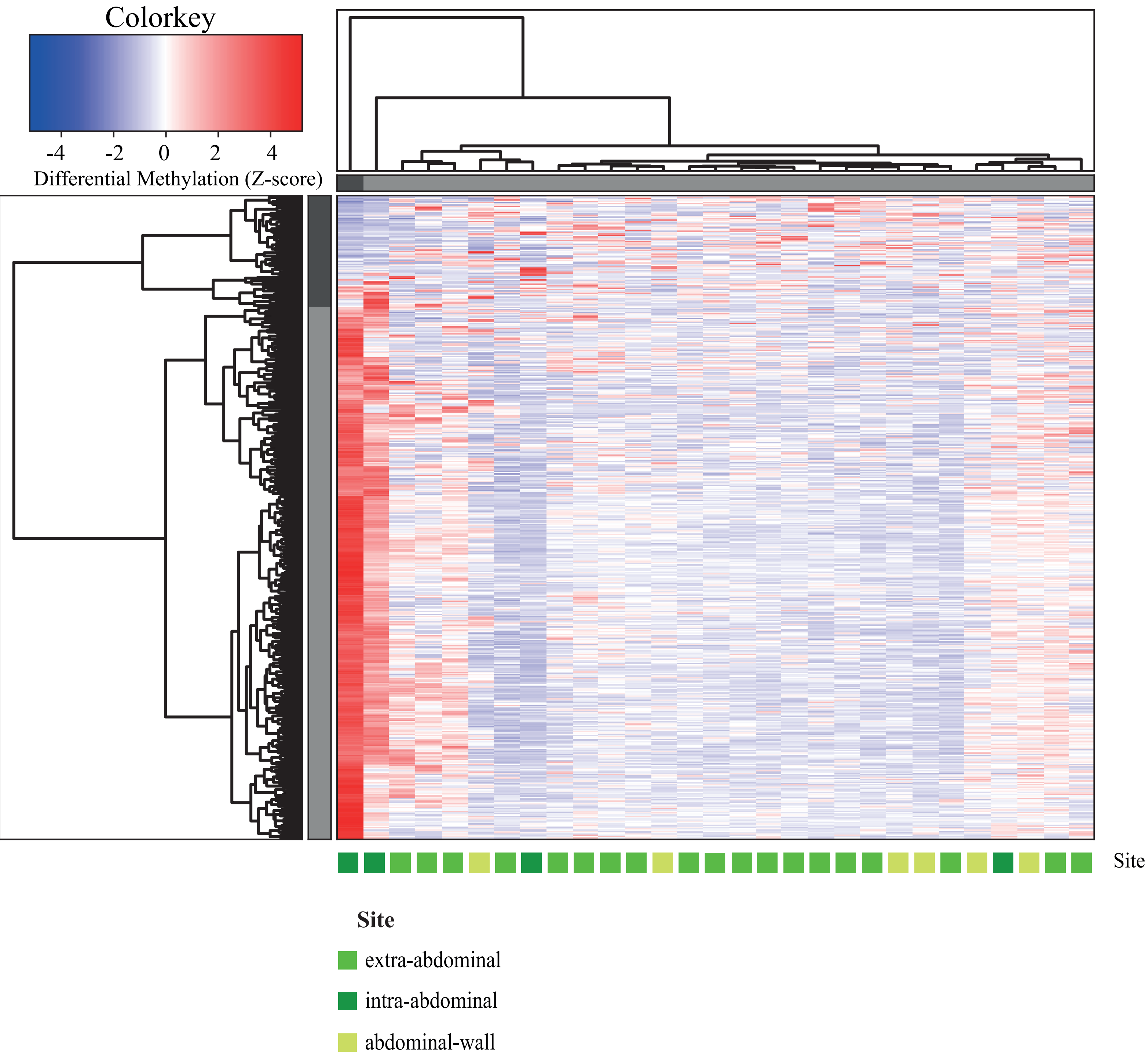

Supplemental Figure 1. Supervised hierarchical clustering based on differentially methylated regions (DMRs) between DTF tumor locations. DTF samples were obtained from tumours at extra-abdominal sites, intra-abdominal sites or the abdominal wall.

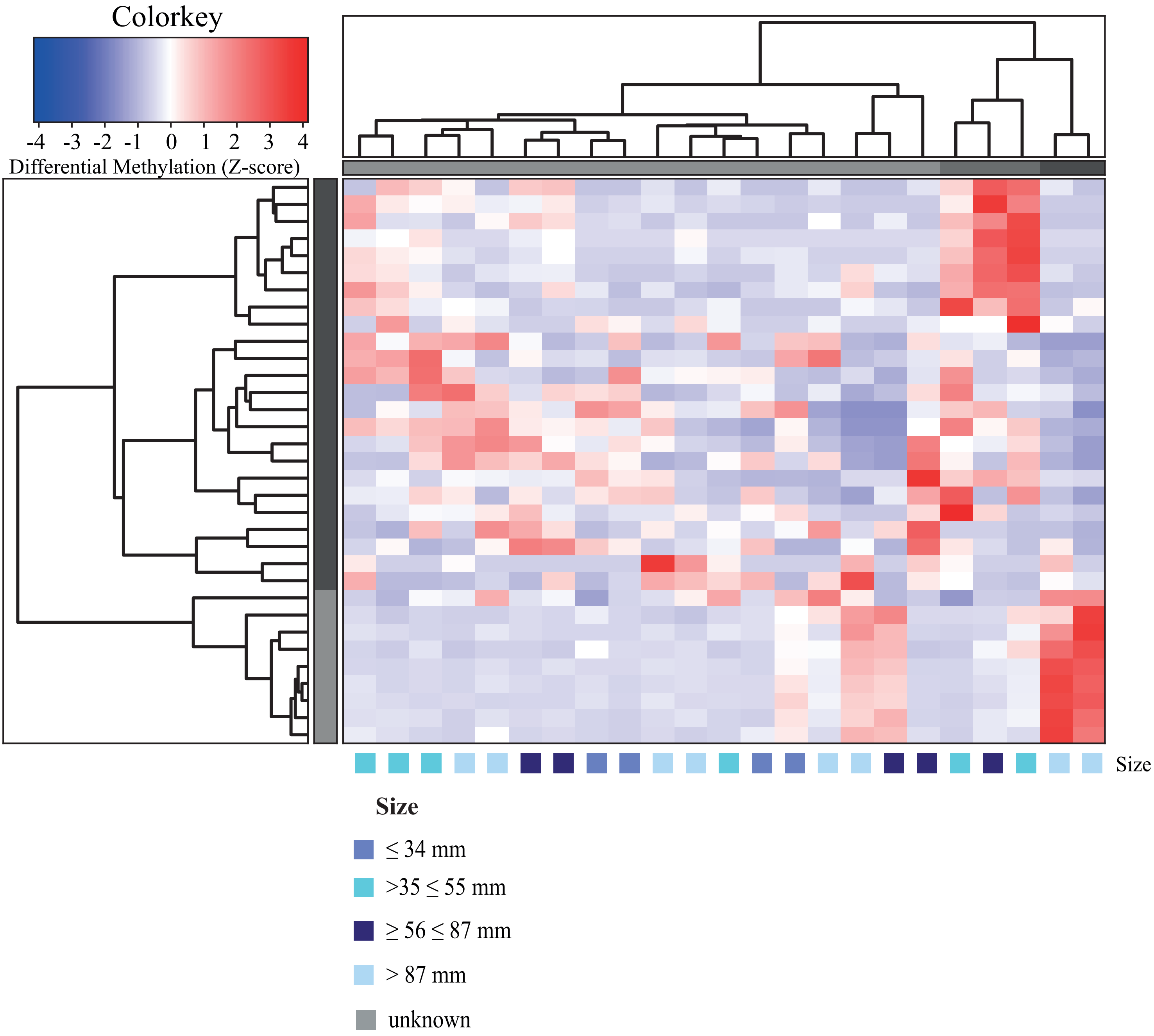

Supplemental Figure 2. Supervised hierarchical clustering based on differentially methylated regions (DMRs) between DTF tumour sizes. Tumour sizes were based on initial imaging data obtained at diagnosis. The following size classes were analysed ≤ 34 mm; >35mm ≤ 55 mm; ≥56 mm ≤ 87 mm; > 87 mm.

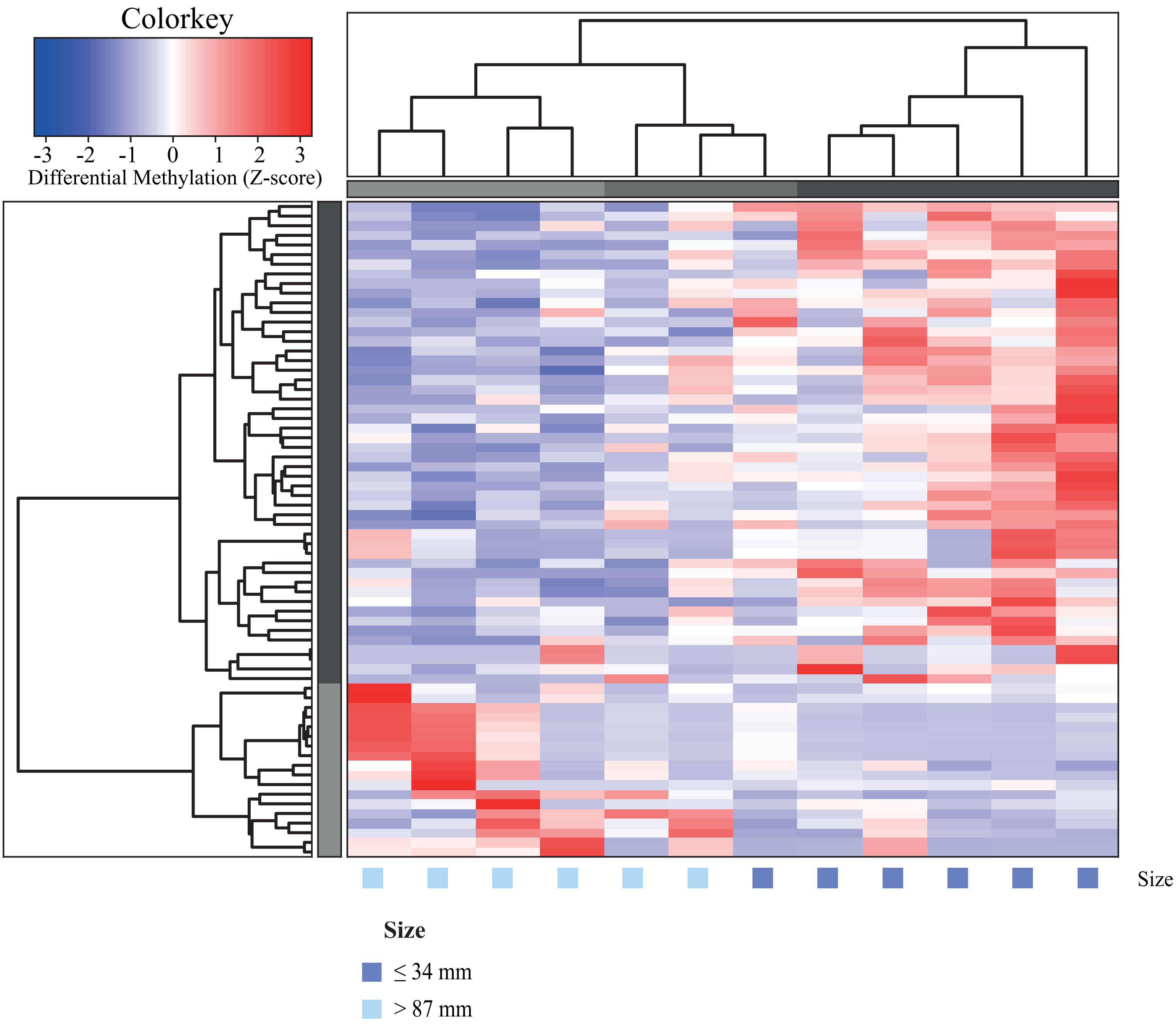

Supplemental Figure 3. Supervised hierarchical clustering based on differentially methylated regions (DMRs) between DTF tumour size extremes. Tumor sizes were based on initial imaging data obtained at diagnosis. The smallest tumours ≤ 34 mm were compared to the largest tumours > 87 mm.

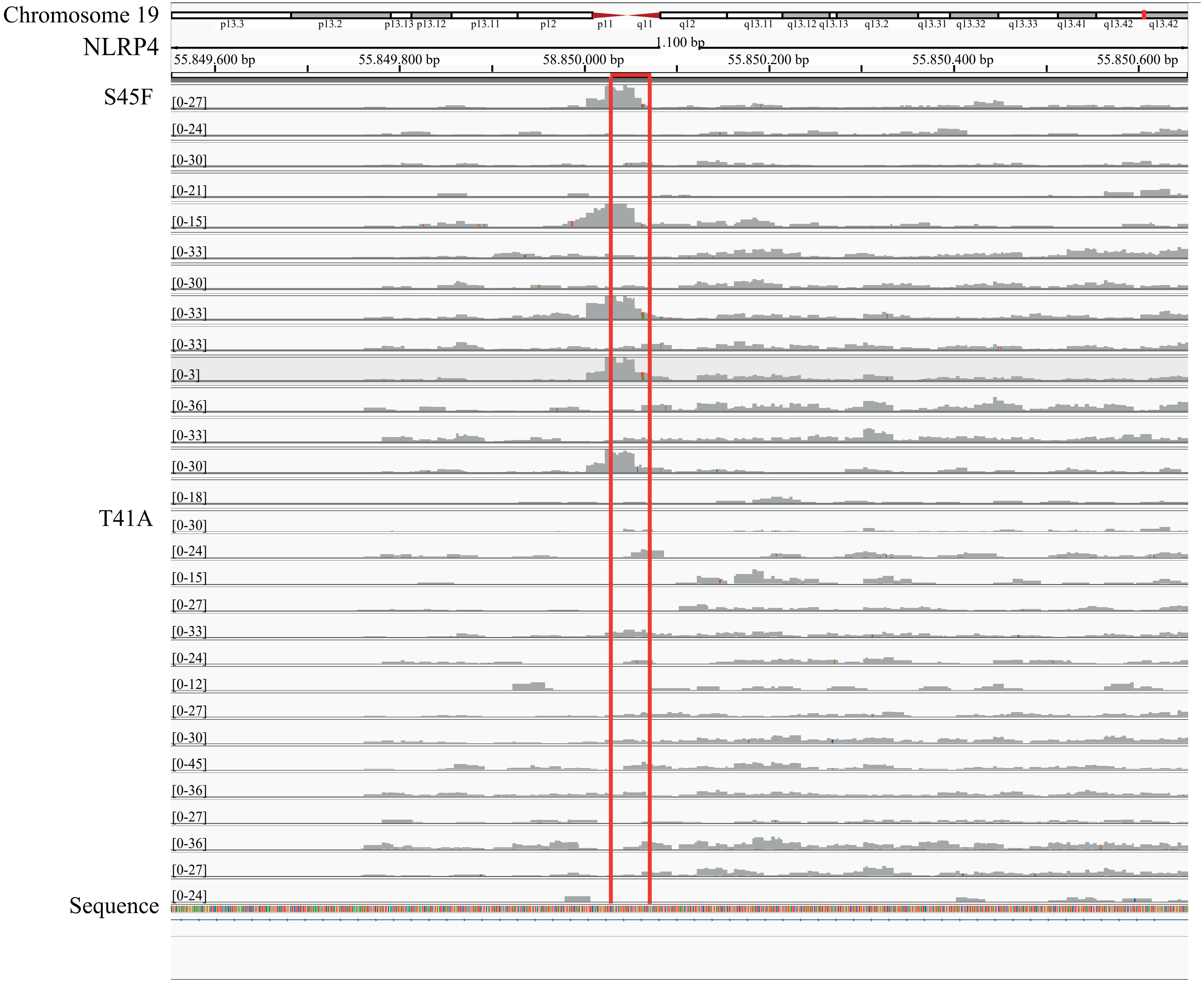

Supplemental Figure 4A. Quantitative visualization of the differentially methylated regions with a fold-change ≥ 1.5 between S45F and T41A DTF. The DMRs are associated with NLRP4. In the top panel, the chromosome involved with the location of the gene indicated by a small red block is depicted. The panels below represent the read counts found in either the S45F or T41A samples with the red box designating the location of the DMR.

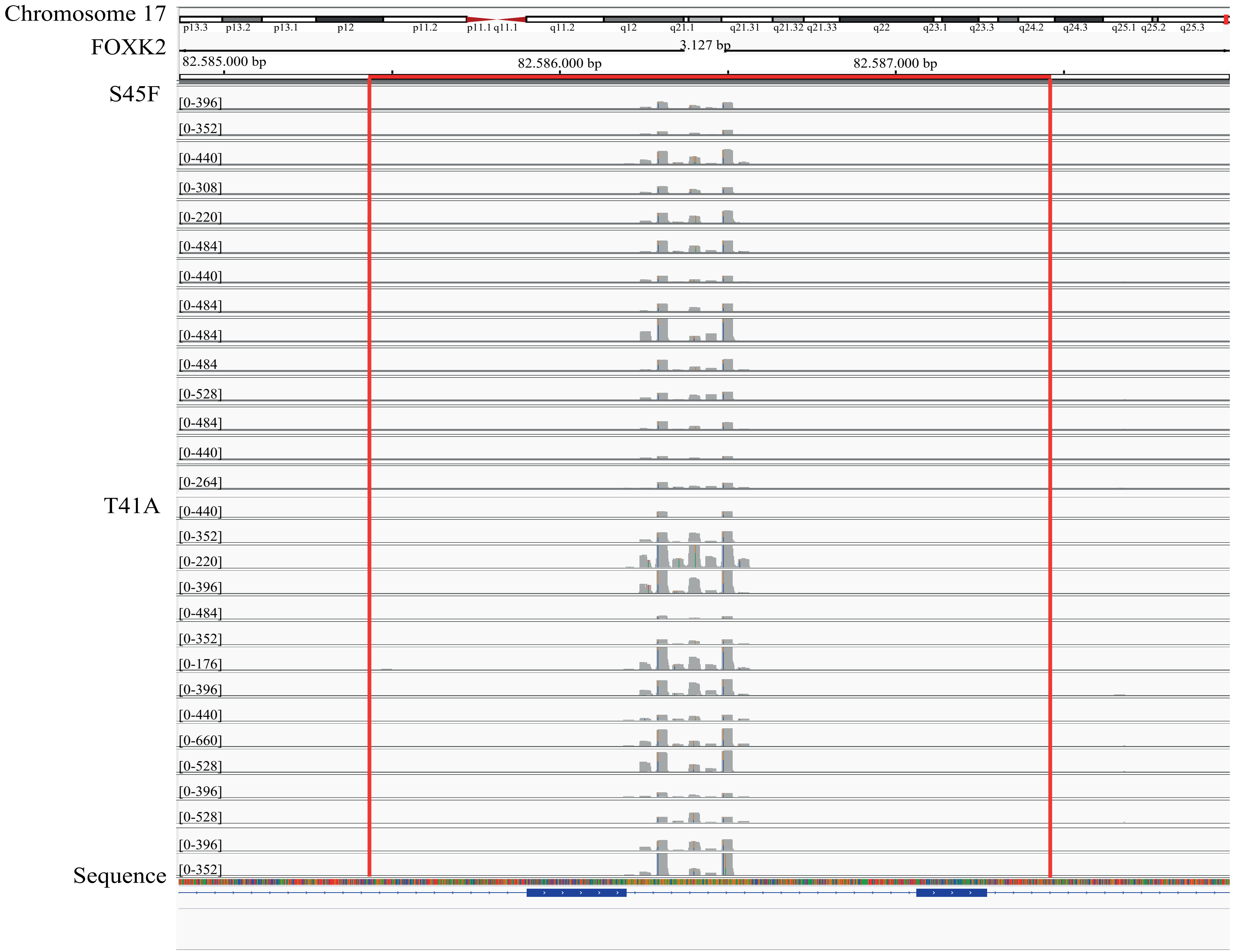

Supplemental Figure 4B. Quantitative visualization of the differentially methylated regions with a fold-change ≥ 1.5 between S45F and T41A DTF. The DMRs are associated with FOXK2. In the top panel, the chromosome involved with the location of the gene indicated by a small red block is depicted. The panels below represent the read counts found in either the S45F or T41A samples with the red box designating the location of the DMR.

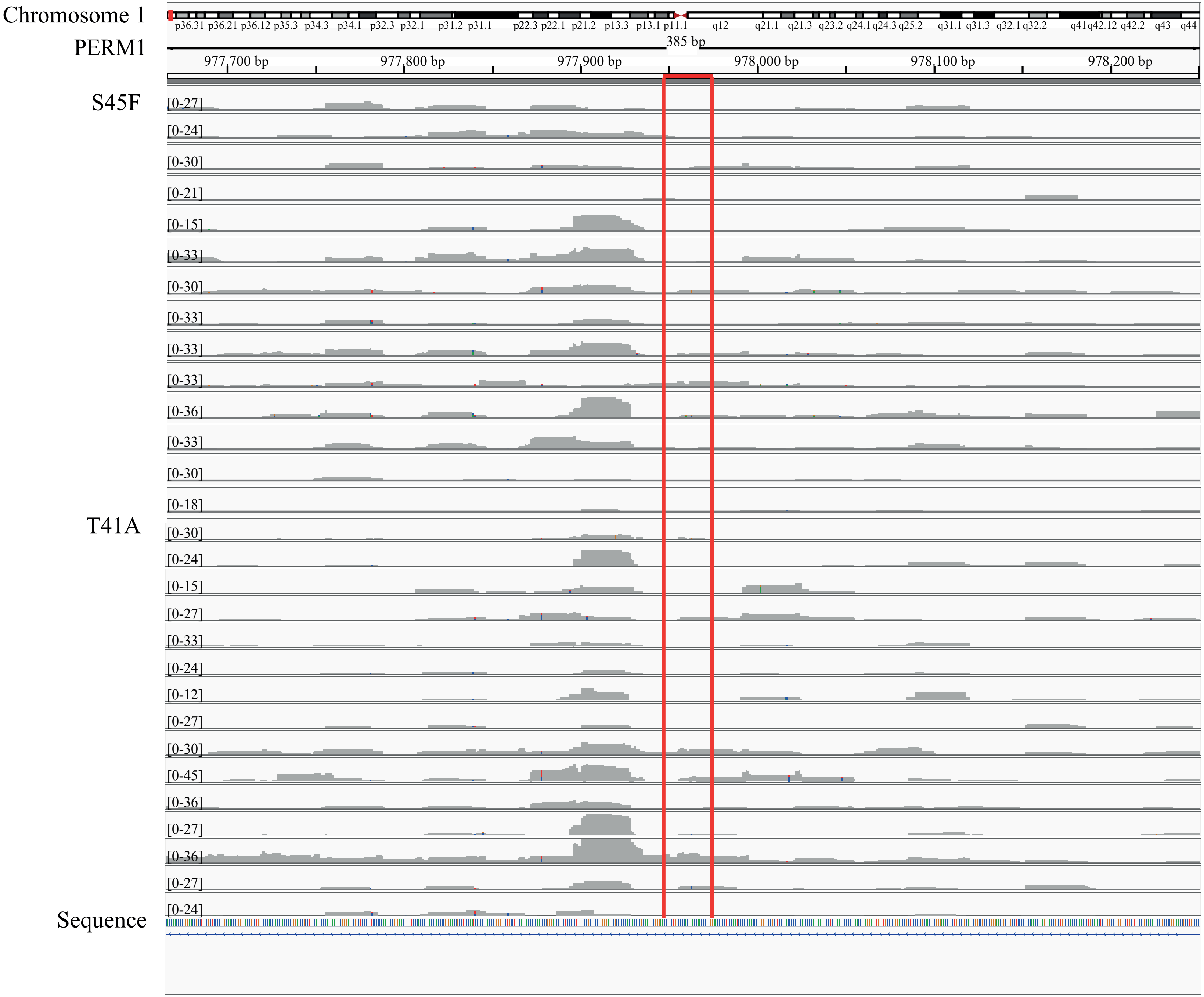

Supplemental Figure 4C. Quantitative visualization of the differentially methylated regions with a fold-change ≥ 1.5 between S45F and T41A DTF. The DMRs are associated with PERM1. In the top panel, the chromosome involved with the location of the gene indicated by a small red block is depicted. The panels below represent the read counts found in either the S45F or T41A samples with the red box designating the location of the DMR.

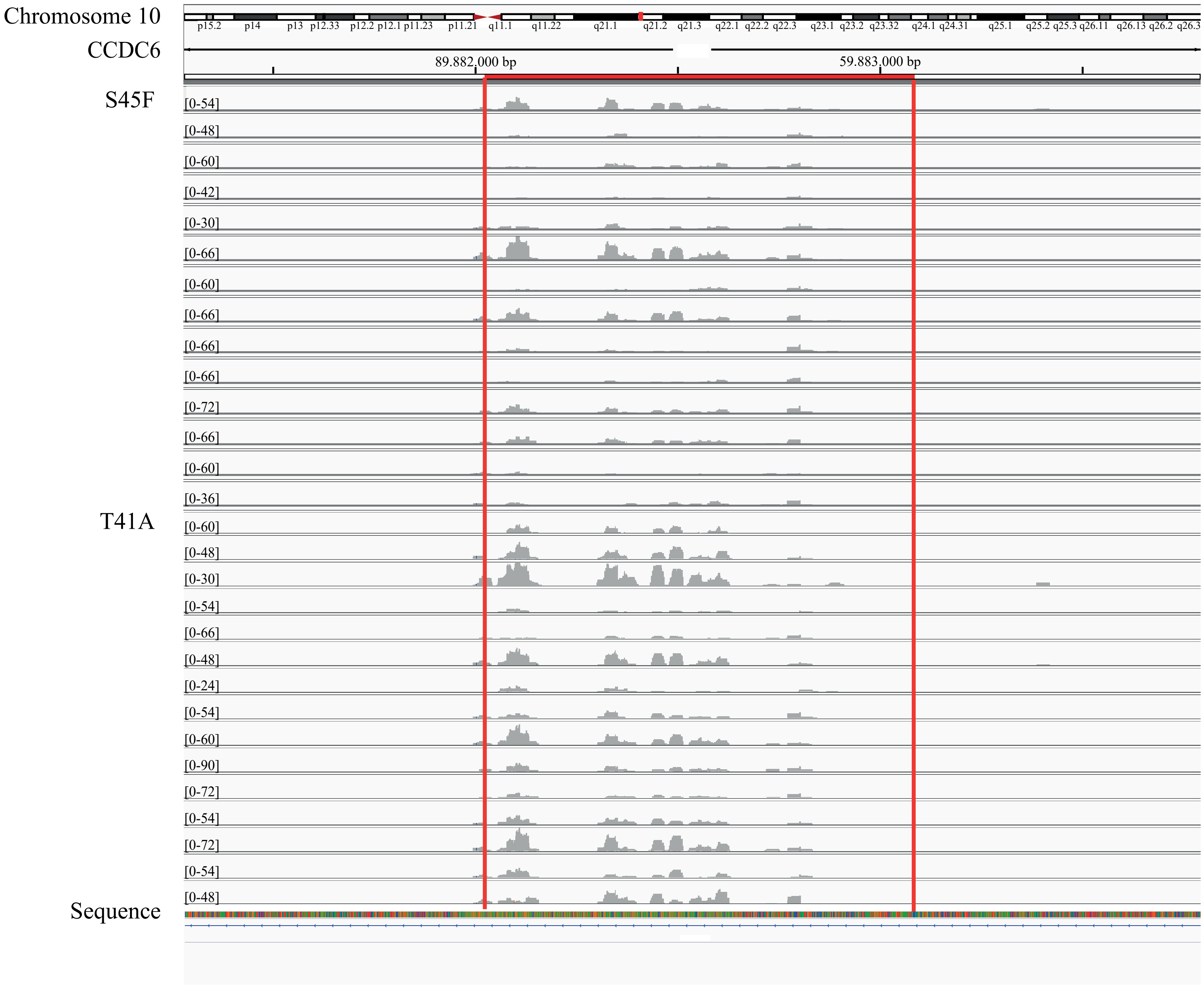

Supplemental Figure 4D. Quantitative visualization of the differentially methylated regions with a fold-change ≥ 1.5 between S45F and T41A DTF. The DMRs are associated with CCDC6. In the top panel, the chromosome involved with the location of the gene indicated by a small red block is depicted. The panels below represent the read counts found in either the S45F or T41A samples with the red box designating the location of the DMR.

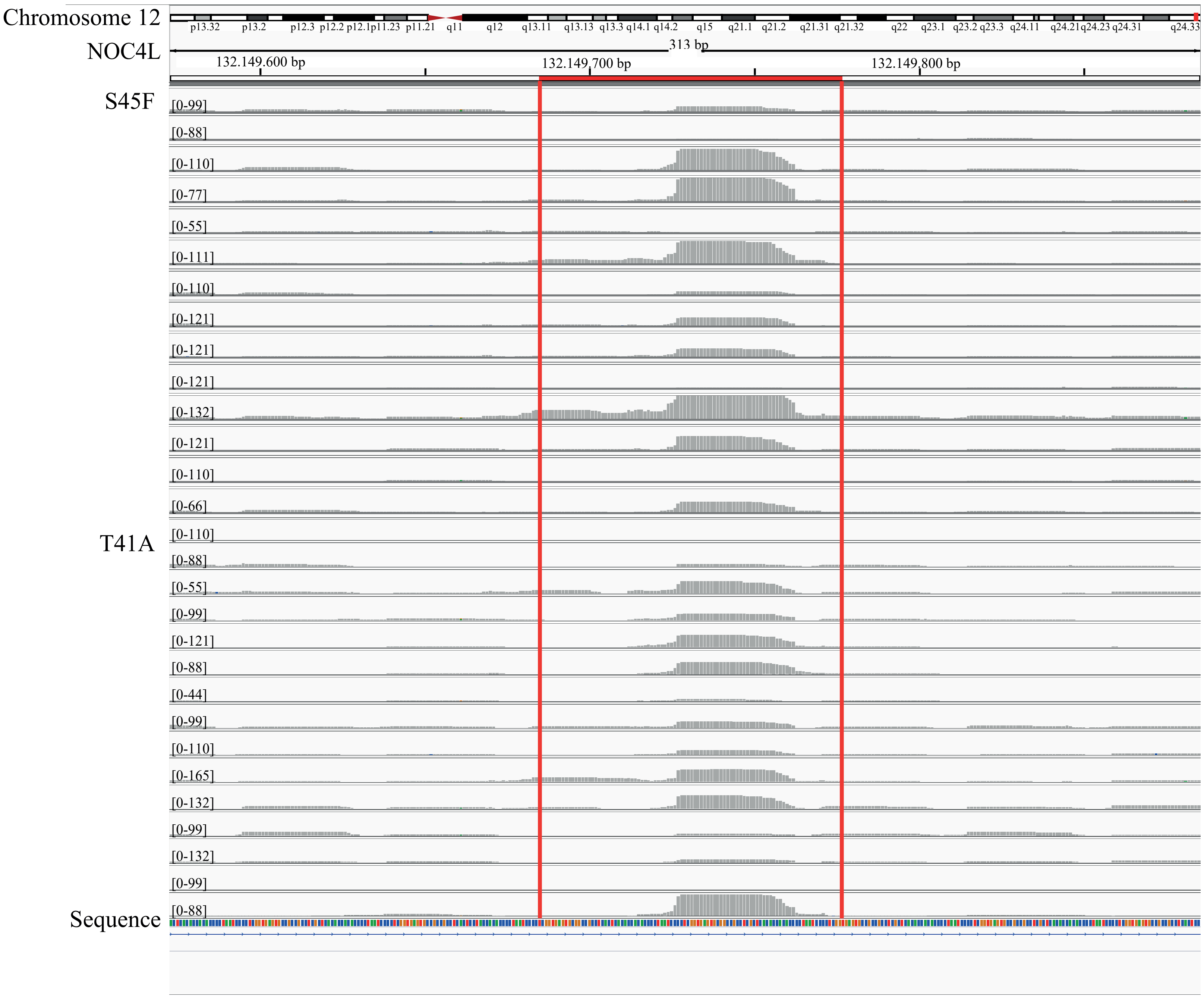

Supplemental Figure 4E. Quantitative visualization of the differentially methylated regions with a fold-change ≥ 1.5 between S45F and T41A DTF. The DMRs are associated with NOC4L. In the top panel, the chromosome involved with the location of the gene indicated by a small red block is depicted. The panels below represent the read counts found in either the S45F or T41A samples with the red box designating the location of the DMR.

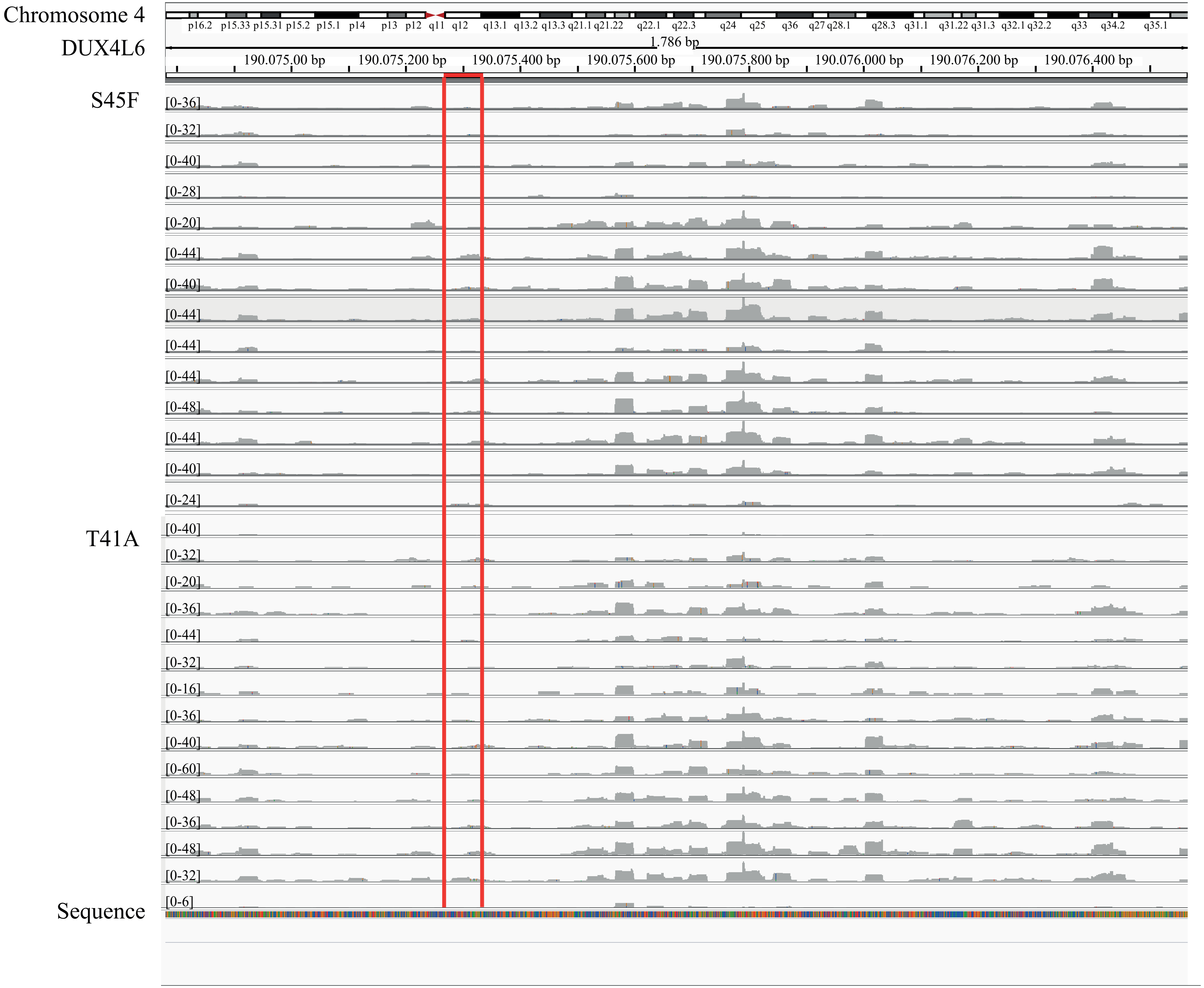

Supplemental Figure 4F. Quantitative visualization of the differentially methylated regions with a fold-change ≥ 1.5 between S45F and T41A DTF. The DMRs are associated with DUX4L6. In the top panel, the chromosome involved with the location of the gene indicated by a small red block is depicted. The panels below represent the read counts found in either the S45F or T41A samples with the red box designating the location of the DMR.

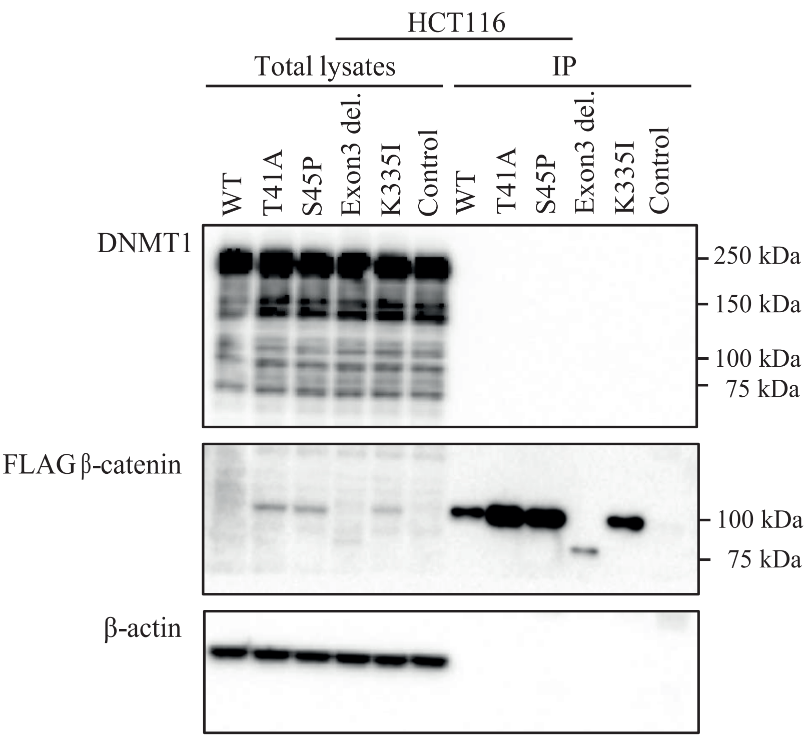

Supplemental Figure 5. DNMT1 is not co-precipitated with wild-type or mutant CTNNB1 (β-catenin). HCT116 cells were transfected with plasmids driving the expression of FLAG-tagged wild-type β-catenin (WT) or FLAG-tagged mutant versions of β-catenin (T41A; S45P; Exon 3 deletion mutant; K335I). As a control cells were transfected with the empty vector. At 48 h post-transfection cell lysates were prepared from which the FLAG-tagged β-catenin variants were immunoprecipitated. Western Blot analysis was used to examine DNMT1, β-catenin and β-actin protein expression in the total lysates and immunoprecipitates.
